# Protein secretion routes in fungi are predicted by the length of the hydrophobic helix in the signal sequence

**DOI:** 10.1093/g3journal/jkag134

**Published:** 2026-07-10

**Authors:** Tristan Sones-Dykes, Edward W J Wallace

**Affiliations:** Institute for Cell Biology and Centre for Engineering Biology, School of Biological Sciences, The University of Edinburgh, Edinburgh EH9 3BF, Scotland, United Kingdom; Institute for Cell Biology and Centre for Engineering Biology, School of Biological Sciences, The University of Edinburgh, Edinburgh EH9 3BF, Scotland, United Kingdom

**Keywords:** signal peptide, translocon, secretion, fungi

## Abstract

Secreted proteins are translocated across membranes through multiple routes. In eukaryotes, secreted proteins with N-terminal signal sequences can use either the signal recognition particle and its receptor or the alternative Sec complex to cross the endoplasmic reticulum membrane. Large-scale experiments on the substrates of these pathways are primarily from the model yeast *Saccharomyces cerevisiae*, but less is known about conservation of translocation pathways. Here, we take a computational approach to analyze secretion signals across the fungal kingdom. Computational predictions by the Phobius model separate secreted proteins in diverse fungal species into distinct populations: cleaved signal peptides with short hydrophobic helices of 8 to 13 amino acids and transmembrane proteins with long hydrophobic helices of 16 to 27 amino acids, similarly to *S. cerevisiae*. These computational predictions also robustly distinguish translocation routes in *S. cerevisiae*: Sec-dependent translocation of native proteins is accurately predicted by the presence of a cleaved signal peptide, while conversely signal recognition particle–dependent translocation is predicted by a retained signal-anchor. Analysis of multiple hydrophobicity scales and signal peptide prediction algorithms shows that the Phobius-predicted length of the hydrophobic helix alone is an effective predictor of translocation route. Our results support the hypothesis that the Sec complex is critical for cell wall biogenesis and protein secretion across fungi.

## Introduction

Protein secretion across cell membranes is critical for cellular life. In fungi, secreted proteins include cell wall proteins that provide a scaffold to construct the cell surface; extracellular secreted proteins such as digestive enzymes that allow fungi to feed from their environment; transmembrane channels and enzymes; and secreted virulence factors and effectors in fungal pathogens of animals, plants, and other fungi (Ellis et al. 2007; Brunke et al. 2016; Gow and Lenardon 2022). Fungal protein secretion is also exploited when using fungi as protein production platforms (Conesa et al. 2001). Understanding the mechanisms of fungal secretion is therefore critical to understanding the fungal lifestyle, fungal pathogenesis, and biotechnological applications. More generally, secreted proteins are crucial for all eukaryotic pathogens (Haldar et al. 2006). However, detailed functional investigations of secretion mechanisms in eukaryotes have focused on a handful of model yeasts and mammalian cells, revealing unexpected complexity (Aviram and Schuldiner 2017). There is now an unmet need to better understand secretion mechanisms in nonmodel eukaryotes.

Secreted proteins integrate into or translocate across lipid membranes to reach their final destination, during or after being translated by cytoplasmically located ribosomes (Rapoport 2007). In eukaryotes, secreted proteins are first translocated across the endoplasmic reticulum (ER) membrane into the ER lumen. Here, we use “secreted” to refer to proteins that are translocated across the ER membrane, whether their final destination is intracellular, the cell surface, or extracellular, and whether or not the mature protein is retained in a membrane. The signal hypothesis states that protein secretion is specified by the protein sequence itself, in the form of an N-terminal signal sequence that directs the protein to a specific destination (Blobel and Sabatini 1971; Blobel et al. 1979).

There are 2 kinds of ER-targeted signal sequences: A signal peptide can be cleaved after the protein is translocated, or retained as a “signal-anchor” that first directs the protein to the ER membrane and then anchors the protein as a transmembrane helix (Martoglio and Dobberstein 1998). Both kinds of signal sequences include hydrophobic helices, either as an “h-region” in a cleaved signal peptide or a transmembrane helix in a signal-anchor (Martoglio and Dobberstein 1998). Compared to transmembrane helices, the h-regions of cleaved signal peptides are (von Heijne 1990) and/or less hydrophobic (Ng et al. 1996).

Differences in signal sequence properties lead to their translocation across the ER membrane by distinct pathways (Ast and Schuldiner 2013). The signal recognition particle (SRP) was discovered in the 1980s (Walter et al. 1981) as an RNA-protein complex, conserved in all domains of life, that binds a signal peptide as it emerges from a ribosome and interacts with a “translocon”—a transmembrane channel that permits the unfolded protein to cross the membrane co-translationally (Pool 2005). The SRP and its receptor target proteins with highly hydrophobic peptides and transmembrane domains (Hessa et al. 2005; Ast et al. 2013; Jan et al. 2014; Costa et al. 2018). However, the SRP does not recognize all signal peptides: In *Saccharomyces cerevisiae*, less hydrophobic signal peptides use an alternative Sec complex, consisting of the Sec61 channel and hetero-tetrameric Sec62/Sec63 complex (Sec62, Sec63, Sec71, Sec72) (Ng et al. 1996). Sec-dependent—i.e. SRP-independent—proteins in *S. cerevisiae* constitute the majority of secreted proteins (Ast et al. 2013). The SRP and Sec pathways share a core channel, called Sec61/SecY, that interacts with different proteins to translocate alternative substrates (Aviram and Schuldiner 2017; Rapoport et al. 2017).

Here, we asked whether signal peptide properties and translocation routes vary across fungi by undertaking a computational analysis of signal peptide sequences. We applied the Phobius algorithm (Käll et al. 2004) to diverse fungi, finding that an overall bimodal distribution of signal sequence features is conserved across fungi. Then we evaluated in silico predictors to classify translocation routes, using *S. cerevisiae* experimental data as ground truth. We found 2 outputs of the Phobius algorithm to be equally good at classifying protein sequences into SRP-dependent or Sec-dependent signals: 8 to 13 amino acid hydrophobic regions are mostly Sec-dependent and are cleaved, while longer 16 to 27 amino acid regions are SRP-dependent and are retained. Two alternative prediction algorithms, DeepTMHMM and SignalP 6.0, also effectively classify the translocation pathway of N-terminal signal sequences. Our analysis suggests that across the fungi, cleaved signal peptides with short hydrophobic helices are likely to target proteins to the Sec-dependent translocation pathway.

## Materials and methods

All data, scripts, and analyses underlying the analysis figures are in the public data repository (https://github.com/TristanSones-Dykes/TMSP_Pub/, doi:10.5281/zenodo.19801296). For managing signal peptide prediction data and website interactions, we used Python and the packages NumPy (Harris et al. 2020), pandas (McKinney 2010), and MechanicalSoup (Moy and Hemberger). Data analysis and visualization made extensive use of R statistical software (R Core Team 2023), including R packages tidyverse and ggplot2 (Wickham et al. 2019), cowplot (Wilke 2025), GGally (Schloerke et al. 2025), and ggtree (Yu et al. 2017).

### Data sources

To predict N-terminal secretion signals with Phobius, we first downloaded the initial 60 amino acids of all annotated protein sequences for selected fungi from FungiDB release 66 in December 2023 (Basenko et al. 2018). These were *S. cerevisiae* S288C; *Candida albicans* SC5314; *Neurospora crassa* OR74A; *Pyricularia oryzae* BR32; *Zymoseptoria tritici* IPO323; *Aspergillus fumigatus* Af293; *Schizosaccharomyces pombe* 972h-; *Puccinia graminis f. sp. tritici* CRL 75-36-700-3; *Ustilago maydis* 521; *Cryptococcus neoformans var. grubii* H99; *Rhizopus delemar* RA 99-880; and *Batrachochytrium dendrobatidis* JEL423. We additionally downloaded full-length protein sequences for selected species from Fungi DB. Human proteins were *Homo sapiens* GRCh38.p14 human_ref proteins, downloaded from NCBI (2012). The protein sequences used are in our public data repository, in the/data/proteins directory.

As a ground-truth experimental dataset, we used a list of *S. cerevisiae* proteins predicted to be translocated into the ER, and not to mitochondria, from Table S1 of Ast et al. (2013). This list of 1,145 proteins includes annotations of verified translocation routes, derived from Ast et al.'s microscopy analysis of mislocalization of *S. cerevisiae* GFP-tagged proteins in a sec72Δ strain compared to a wild-type strain, as described in detail in the original paper (Ast et al. 2013). Sec-dependent proteins (called SRP-independent in Ast et al. Table S2) are those whose localization changes in sec72Δ, while SRP-dependent (called Sec72-independent in Ast et al. Table S3) are those whose localization does not change in sec72Δ.

### Signal peptide prediction

We applied the Phobius algorithm (Käll et al. 2004) to each species proteome dataset on the Phobius server at https://phobius.sbc.su.se/, after trimming annotated proteins to exactly 60aa in length. The Phobius predictions were processed to create a .csv file with the start and end of the hydrophobic region (signal peptide h-region or transmembrane helix region) for each protein with a predicted secretion signal.

We applied the DeepTMHMM algorithm (Hallgren et al. 2022) to all annotated *S. cerevisiae* full-length proteins. Then, we classified each protein by testing which had predicted signal peptides and/or transmembrane regions in the initial 60 amino acids. Of those, we took a final subset using those that had no more than 2 predicted regions (SP/TM) and calculated the final window length as the sum of their lengths. This was required because DeepTMHMM is not restricted to classifying a single region per protein and can predict multiple same-type regions per protein. DeepTMHMM region predictions have no lower bound on length—often predicting multiple windows of 2 or 3 amino acids—so we used this method to reduce the predictive noise from the algorithm. Summary counts of the DeepTMHMM classifications can be found in the deeptmhmm_results.md files in the results folder of the repository.

We applied SignalP6.0 algorithm (Teufel et al. 2022) (in slow sequential mode) to all annotated *S. cerevisiae* full-length proteins.

### Data analysis and visualization

We constructed contingency tables to test how well each classifier (signal sequence length and hydrophobicity, signal peptide vs transmembrane region, etc.) predicted verified translocation route in *S. cerevisiae*. These contingency tables contain 4 counts in 2 × 2 format, i.e., (classifier outcomes) × (Sec-dependent or SRP-dependent). For each contingency table, we ran a χ² independence test with the null hypothesis that these 2 categorical variables (predicted, verified) are correlated—precisely, Pearson's χ² test with Yates’ continuity correction using the χ² test function in R. We applied the same contingency table analysis and χ² test to test if predictions of alternative algorithms were in agreement.

For the classification based on signal sequence length, we fit a Gaussian mixture model, using the normalmixEM function from the mixtools library (Benaglia et al. 2009), on our *S. cerevisiae* predicted proteins. The intersection of the 2 Gaussian components—between 13 and 14 amino acids—was used as our classification boundary for the rest of the analysis.

We performed Gene Ontology (GO) analysis using FungiDB, release 66 (Basenko et al. 2018), with default settings for cell component terms, on each fungal species separately.

## Results

### Signal sequence features are bimodally distributed across fungal species

If all fungi have similarly distributed signal sequence features, this would support a model of conserved properties and processing across fungi, even in the absence of direct experimental measurements. So, we predicted signal sequences and lengths of their hydrophobic helices in a diverse selection of other fungi spanning approximately 1 billion years of fungal evolution (Li et al. 2021). These fungi include model organisms, important pathogens, and industrially relevant fungi: ascomycetes (*S. cerevisiae*, *C. albicans*, *Pichia pastoris* aka *Komagataella phaffii*, *N. crassa*, *Trichoderma reesei*, *Magnaporthe grisea*, *Z. tritici*, *A. fumigatus*, *S. pombe*), basidiomycetes (*P. graminis*, *U. maydis*, *C. neoformans*), and earlier-diverging mucorales (*R. delemar)* and chytrids (*B. dendrobatidis*). We downloaded protein sequences from FungiDB (Basenko et al. 2018). We also extended the analysis to *H. sapiens* as an outgroup.

We initially analyzed signal sequences with the Phobius software (Käll et al. 2004). Phobius predicts cleaved signal peptides and N-terminal transmembrane helices based on a hidden Markov model, which includes predictions of subregions of cleaved signal peptides: the n-region, hydrophobic h-region, and c-region up to the cleavage site. Phobius was prioritized because its performance has been validated on ground-truth data in *S. cerevisiae* (Ast et al. 2013), the analysis that we sought to extend to other fungi.

**Fig. 1.**
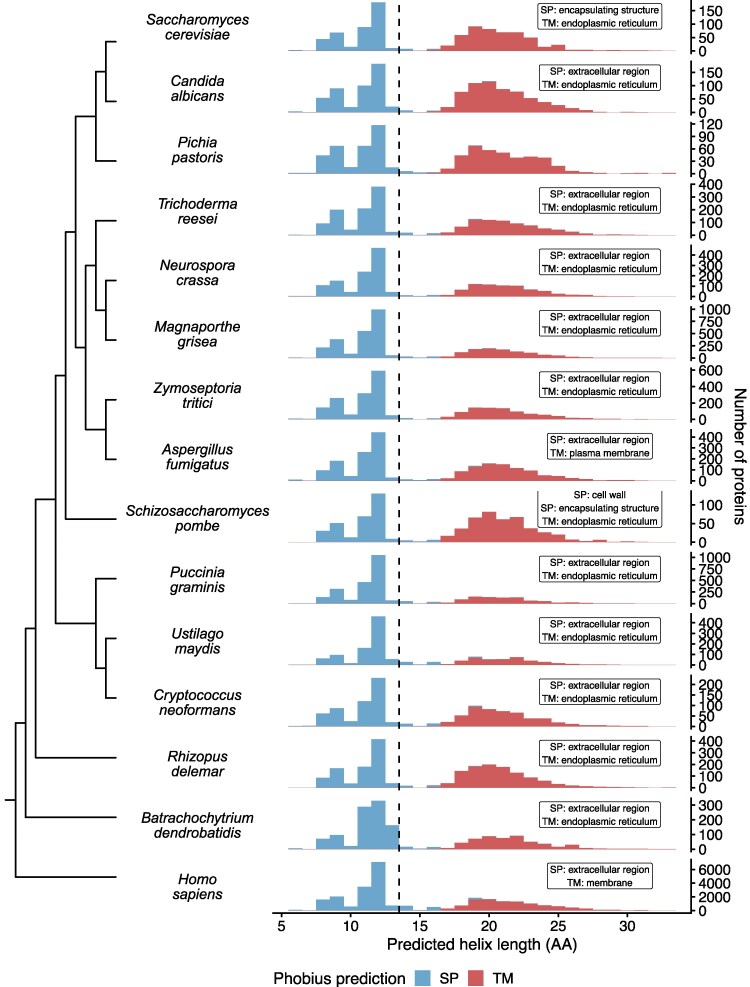
Predicted hydrophobic helices in signal peptides have a bimodal length distribution across fungi. Left panel shows cladogram of 14 included fungal species and outgroup *H. sapiens*; right panels show a histogram of the number of proteins with each helix length predicted by Phobius, colored as the h-region of a cleaved signal peptide (SP) or as the transmembrane helix of a retained signal-anchor (TM). The dashed line indicates a threshold at 13 amino acids. The *y* axis scales are different in each panel, reflecting differences in the total number of secreted proteins between species. The inset text shows the GOSlim term with the lowest *P*-value for each predicted group, except for *S. pombe* where 2 terms had the same *P*-value and *Pichia pastoris* (*K. phaffii*) where GO annotations were not adequate. We abbreviated “external encapsulating structure” to “encapsulating structure”.

Signal sequence properties are similar across fungi: Each has a bimodal distribution of helix lengths, with almost all predicted cleaved signal peptides having h-regions of length 8 to 13 and almost all predicted N-terminal transmembrane helices having length 16 to 27 (Fig. 1). In particular, the bimodal distribution of predicted hydrophobic helix lengths in *S. cerevisiae* is representative of other fungi. The number of proteins in each category varies widely; for example, *P. graminis* has roughly 10 times the number of proteins with cleaved signal peptides as *S. pombe*. There are some minor differences in the distribution, for example, the early-diverging chytrid *B. dendrobatidis* has more predicted h-regions of lengths 11 and 13 relative to length 12, which could indicate differences in preferred h-regions. Surprisingly, Phobius predicts very few h-regions of predicted length exactly 10 in any species. Overall, the conserved bimodal distributions are consistent with cleaved signal peptides with short hydrophobic helical h-regions being categorically different from retained signal-anchors that have longer transmembrane regions.

To understand the functional properties of proteins with predicted cleaved signal peptides or signal-anchor transmembrane regions, we performed a GO analysis using FungiDB (Basenko et al. 2018). As expected, across fungi, proteins with predicted cleaved signal peptides are enriched in GO terms for cell wall (GO:0005618), external encapsulating structure (GO:0030312), and extracellular region (GO:0005576). Both cleaved signal peptides and predicted transmembrane regions are enriched for GO terms for ER (GO:0005783), plasma membrane (GO:0005886), and in some cases vacuole (GO:0005773) and Golgi apparatus (GO:0005794). In all cases, the *P*-values for GO enrichment are below 10^−10^. This analysis supports conservation of protein functions within each category, despite the limits of GO analysis in nonmodel fungi.

Having established that 2 classes of predicted signal sequences are consistent across fungi, we next sought to use *S. cerevisiae* experimental data to ask if this classification of sequences could correspond to the classification of translocation pathways.

### Hydrophobic helix length in signal regions predicts translocation route in *S. cerevisiae*

To evaluate signal peptide translocation route predictions in *S. cerevisiae*, we re-analyzed ground-truth experimental data curated by Ast et al. (2013). They classified secreted proteins into 3 groups: “Sec-dependent” (called SRP-independent by Ast et al.) based on mislocalization in sec72Δ cells, “SRP-dependent” based on unperturbed localization in sec72Δ, and “unverified” where no clear experimental result is available. Ast et al. (2013) compared this experimental data with computational predictions of a curated list of 1,145 *S. cerevisiae* proteins that are predicted to be translocated into the ER and analyzed their N-terminal 60 amino acids (aa) using Phobius [25]. We likewise applied Phobius to the initial 60 amino acids of all annotated *S. cerevisiae* proteins, to generate a list of 925 proteins with predicted secretion signals within the N-terminal 60aa. Thus, every protein on this list has a predicted hydrophobic helix near the N-terminus, either as the h-region of a cleaved signal peptide or as a retained transmembrane helix. We compared both the length of the predicted hydrophobic helix and the maximum hydropathy of 9-aa windows on the Kyte-Doolittle scale (Kyte and Doolittle 1982). This 9-aa window length was verified thoroughly in Ast et al. (2013).

**Fig. 2.**
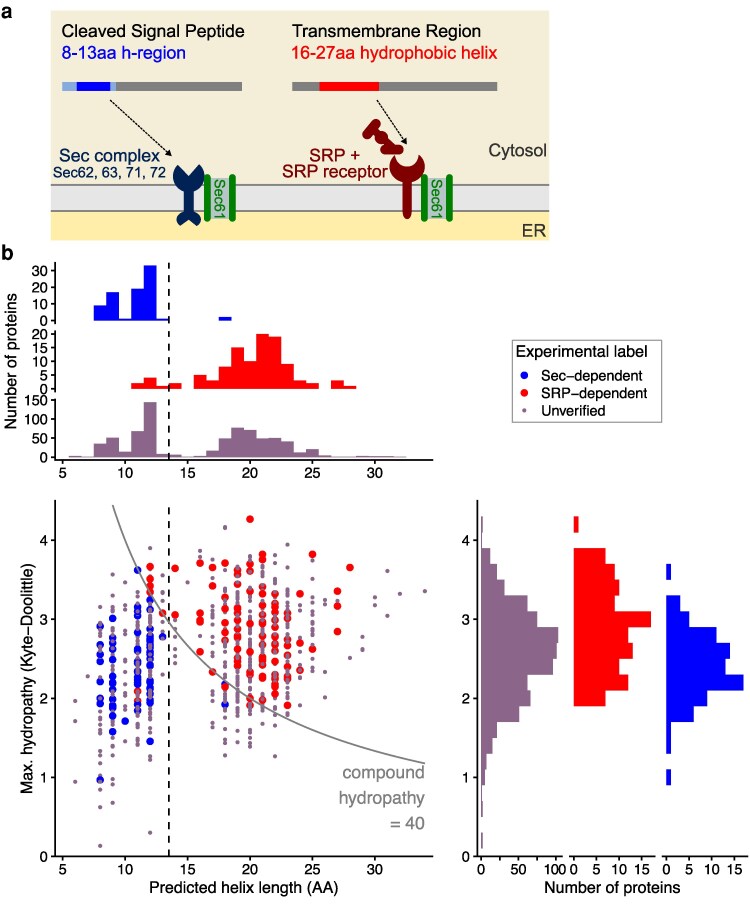
Hydrophobic helix length in signal regions predicts translocation route in *S. cerevisiae*. a) Model of secreted proteins where cleaved signal peptides that have short hydrophobic helices (h-regions) are generally Sec-dependent, while those with N-terminal transmembrane helices are generally SRP-dependent. The Sec complex comprises the Sec61 channel with Sec62, Sec63, and in fungi Sec71 and Sec72; the SRP pathway involves the SRP, and the SRP receptor with the Sec61 channel. b) The main panel is a scatter plot of predicted secreted proteins, showing predictions of helix length from Phobius compared to the maximum hydropathy in a 9-aa window on the Kyte-Doolittle scale. The black dashed line separates Sec-dependent from SRP-dependent proteins by length; the gray line separates by compound hydropathy score of 40 as in Ast et al. (2013). Marginal distributions are shown: The top plot is a histogram showing the numbers of proteins by helix length, which is bimodal, and the right plot by maximum hydropathy, which is unimodal.

Predicted hydrophobic helices show a bimodal distribution of helix lengths, with each mode corresponding to one experimentally validated translocation route (Fig. 2). Verified Sec-dependent proteins have predicted 8 to 13 amino acid helices while SRP-dependent proteins have predicted 16 to 27 amino acid helices. Unverified proteins also have a bimodal distribution. Of 82 verified Sec-dependent proteins, 80 have helix length 13 or less while 96 of the 107 SRP-dependent proteins have helix length of 14 amino acids or more (dashed vertical line in Fig. 2). Applying a χ² test to this contingency table, we found that the length of the predicted helix alone can be used to classify proteins into Sec-dependent or SRP-dependent (*P* < 10^−30^). Similarly, we constructed a contingency table of Phobius predictions for cleaved signal peptides or signal-anchor transmembrane helices, compared to verified Sec-dependent or SRP-dependent categories; a χ² test on this contingency table also finds the prediction to be excellent (*P* < 10^−30^). This bimodality is not forced by the Phobius algorithm, which allows h-regions of length 6 to 20 amino acids within a cleaved signal peptide of indefinite total length, and transmembrane helices of length 15 to 34 amino acids, although of course the h-regions are included in Phobius predictions alongside the other signal peptide regions and the cleavage site (Käll et al. 2004).

By contrast, maximum hydropathy of N-termini has a unimodal distribution (Fig. 2). As previously reported, maximum hydropathy for Sec-dependent proteins is on average lower than that of SRP-dependent proteins (Ast et al. 2013), yet there is considerable overlap in their distributions. This means that maximum hydropathy is not by itself a good predictor of translocation route. Following the idea that overall hydropathy determines translocation route (Ng et al. 1996), Ast et al. computed compound hydropathy scores as the product of hydrophobic helix length and maximum hydropathy in a 9-aa window and showed that these are bimodally distributed (Ast et al. 2013). Repeating their analysis shows that of 82 Sec-dependent proteins all have compound hydropathy under 40, and of 107 SRP-dependent proteins, 94 have compound hydropathy over 40 (solid curve in Fig. 1). A χ² test on this contingency table shows that compound hydropathy is an effective classifier (*P* < 10^−30^). However, hydrophobic helix length is a simpler metric and an equally effective classifier.

The above analysis is limited by using only one hydrophobicity scale (Kyte-Doolittle) and one prediction algorithm (Phobius) to assess signal sequence properties.

### Amino acid frequencies in hydrophobic helices differ by secretion route

We asked if metrics beyond Kyte-Doolittle hydropathy and hydrophobic helix length could help to distinguish between Sec-dependent and SRP-dependent secretion signals. We initially evaluated other hydrophobicity scales to see if they would provide a more effective biologically relevant classifier of translocation route: The Rose hydrophobicity score is defined by the area that a residue buries upon folding in a globular protein (Rose et al. 1985), and the Hessa hydrophobicity score is defined by the contribution of a residue to transmembrane helix insertion into a membrane (Hessa et al. 2005). On average, Sec-dependent and SRP-dependent proteins have different maximum Rose or Hessa hydrophobicity (Supplementary Figs. 1 and 2), and the distributions clearly have distinct means with higher maximum hydrophobicity of SRP-dependent signals. However, we found no meaningful improvements in classification compared to using the Kyte-Doolittle scale or the predicted length of the hydrophobic helix.

**Fig. 3.**
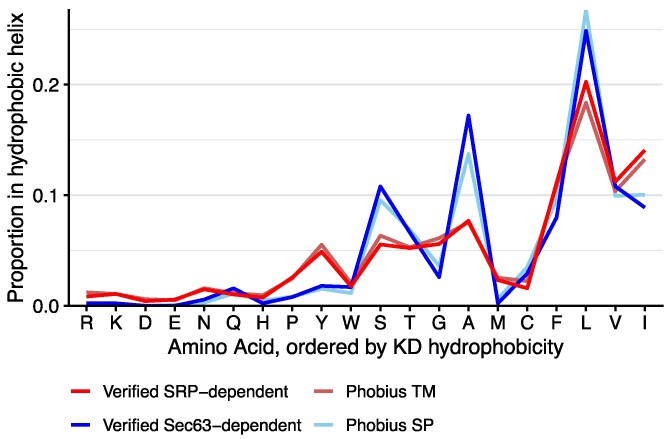
Amino acid frequencies in hydrophobic helices differ between Sec-dependent and SRP-dependent proteins. Plot shows the proportion/frequency of amino acid usage in N-terminal hydrophobic helices of *S. cerevisiae* secreted proteins, either h-regions of cleaved signal peptides (SP) or N-terminal transmembrane helices (TM), as predicted by Phobius. Amino acids are ordered by increasing Kyte-Doolittle (KD) hydrophobicity.

Next, we asked how the amino acid composition of hydrophobic helices differs by verified or predicted secretion route. We compared N-terminal hydrophobic helices of *S. cerevisiae* secreted proteins, using the dataset of 925 proteins analyzed in Fig. 1. We computed the amino acid composition of h-regions for predicted cleaved signal peptides, or of entire predicted transmembrane regions of this set of proteins. We compared 4 groups of proteins: verified Sec-dependent proteins or SRP-dependent proteins, or those with Phobius-predicted cleaved signal peptides or retained transmembrane regions (Fig. 3). Verified SRP-dependent proteins and predicted N-terminal transmembrane regions have similar amino acid composition, particularly enriched in leucine. Sec-dependent h-regions have different frequencies, with relative enrichment of leucine, serine, and alanine and relative depletion of tyrosine. Note the average lengths of these 2 groups differ, so the Sec-dependent proteins have lower amino acid counts overall.

Overall, this analysis of amino acid composition supports the conclusion that h-regions of verified Sec-dependent proteins are typical of the wider set of predicted cleaved signal peptides and likewise that transmembrane regions of verified SRP-dependent proteins are typical of the wider set of predicted N-terminal transmembrane regions. Our attempts at more detailed analysis of position-dependent amino acid frequencies yielded no clear insight beyond the leucine-richness of h-regions as previously reported (Xue et al. 2023). Further insight may be limited by the low absolute numbers of residues per signal peptide, for example, an average of 3 leucine residues across a h-region of length 12, or 1 tyrosine in a transmembrane helix of length 20. These amino acid patterns are likely reflected in the weights embedded in the Phobius algorithm, leading to a risk of circular reasoning, and future computational work will have to address the sequence and structural determinants of translocation route in more detail.

### Comparing signal sequence predictions between algorithms

To check our Phobius-based predictions of secretion route and hydrophobic region length, we evaluated 2 alternative prediction algorithms. DeepTMHMM (Hallgren et al. 2022) is a recent deep neural-network based predictor of transmembrane topology that, like Phobius, separately detects cleaved signal peptides and transmembrane helices. However, DeepTMHMM does not predict separate h-region lengths within the cleaved signal peptide. SignalP6.0 (Teufel et al. 2022) is another recent deep neural-network based predictor of signal peptides that does predict h-region lengths. However, SignalP6.0 does not separately classify TM helices.

In the verified set of *S. cerevisiae* secretome from Ast et al. used above, DeepTMHMM cleaved signal peptide vs transmembrane helix categories also predict Sec-dependent or SRP-dependent secretion: a χ² test on the contingency table of DeepTMHMM predictions compared to verified translocation route finds the prediction to be excellent (*P* < 10^−27^). DeepTMHMM and Phobius mostly predict the same *S. cerevisiae* proteins as having cleaved signal peptides or retained transmembrane helices (Supplementary Fig. 3). When both tools give a prediction, 580 out of 665 of those predictions agree; a χ² test on this contingency table passes (*P* < 10^−35^). However, some proteins are predicted in one tool but not the other, and Phobius predicts a larger number of the verified proteins as secreted.

In the verified set of *S. cerevisiae* secretome from Ast et al. used above, SignalP6.0 predicted cleaved signal peptides also predict Sec-dependent secretion: Of 82 verified Sec-dependent proteins, 76 are predicted by SignalP6.0 while of the 107 SRP-dependent proteins only 6 are predicted by SignalP6.0. A χ² test finds the prediction to be excellent (*P* < 10^−15^), although it is marginally less clear-cut than the Phobius predictions. Applied to the whole *S. cerevisiae* proteome of 5907 verified ORFs, SignalP6.0 and Phobius predict an overlapping set of proteins to contain signal peptides, with 278 in common, 147 called by Phobius and not SignalP6.0, and 19 called by SignalP6.0 and not Phobius. Of the proteins predicted to have SPs by Phobius and not SignalP, there were several annotated as transmembrane proteins, including in the mitochondrial and vacuolar membranes. As disentangling these predictions was not the purpose of our analysis, we did not proceed further.

**Fig. 4.**
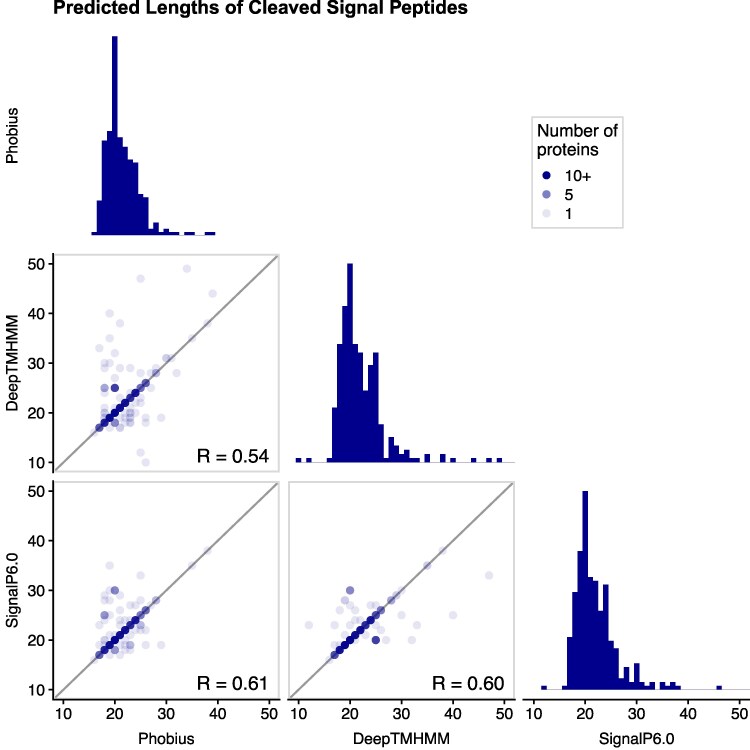
Three computational tools, Phobius, DeepTMHMM, and SignalP6.0, somewhat agree on predicted lengths of cleaved signal peptides. Panels below the diagonal compare the predicted lengths of the entire signal region from *S. cerevisiae* proteins, restricted to *S. cerevisiae* proteins for which any tool predicted such features. For DeepTMHMM, the TM helix had to be predicted in the initial 60 amino acids. The opacity of each bubble is proportion to the count of proteins with that combination of predicted lengths, as shown in the legend. Pearson's *R* correlation coefficients are shown. Panels on the diagonal show the numbers of proteins with each predicted length for each tool as a histogram.

We next compared predictions of signal peptide length in *S. cerevisiae* for Phobius, DeepTMHMM, and SignalP6.0 (Fig. 4). The length of the entire signal peptide, i.e. its length up to the cleavage site, is longer than the h-region and was compared because DeepTMHMM outputs only the overall length of the signal peptide. There is substantial agreement between tools, with many predicted lengths identical between tools (dark bubbles along the diagonal in Fig. 4). However, the overall correlations are moderate (Pearson's *R* = 0.54 to 0.61), because of the number of signal peptides with divergent predicted lengths.

We also compared predictions of signal peptide class for Phobius and DeepTMHMM (Supplementary Fig. 3): Where their predictions agree on category, they somewhat agree on the predicted overall lengths of cleaved signal peptide. However, correlations between predicted lengths of predicted transmembrane helices are weak (Pearson's *R* = 0.19), and indeed some DeepTMHMM helix length predictions are too short to span a membrane. Unsurprisingly, where Phobius and DeepTMHMM predictions disagree on the category, predictions of hydrophobic helix length are uncorrelated.

Lastly, we compared predictions of h-region length of cleaved signal peptides between Phobius and SignalP6.0 (Supplementary Fig. 4). These predictions are only weakly correlated (Pearson's *R* = 0.33), and the predicted h-region length from SignalP6.0 is longer than from Phobius. There are many possible explanations for this, including the possibility that SignalP6.0's deep learning algorithm is predicting an “extended h-region” that extends beyond a core helical h-region.

We conclude that Phobius, DeepTMHMM, or SignalP6.0 predictions of either cleaved signal peptide vs transmembrane helix are effective predictors of translocation route by the Sec complex or the SRP pathway. Predictions of these tools generally agree on category, i.e. the presence of a cleaved signal peptide, but only moderately agree on the length of signal peptides or on the length of h-regions.

## Discussion

### Hydrophobic helix length predicts translocation route of signal peptides in fungi

This work extends and simplifies previous results on how N-terminal sequences specify the route of translocation into the ER. We used curated experimental data in *S. cerevisiae* and validated computational prediction algorithms to show that, overwhelmingly, Sec-dependent proteins have cleaved signal peptides while SRP-dependent proteins have retained transmembrane signal-anchor peptides (Fig. 2).

Previous work has argued that the hydrophobic helices of cleaved signal peptides (h-regions) are either longer (von Heijne 1990; Nielsen et al. 2019) or less strongly hydrophobic (Ng et al. 1996; Ast et al. 2013) than transmembrane regions. Here, our analysis using the Phobius algorithm argues that length is the main predictor: Hydrophobic regions of cleaved signal peptides are than transmembrane helices, in a pattern conserved across fungi (Fig. 1). This is consistent with previous large-scale analysis (Käll et al. 2004; Liaci et al. 2021). Also, in mammals, predicted h-regions have fewer leucines than transmembrane regions (Hessa et al. 2005). By contrast, we found that the maximum hydrophobicity of the predicted signal sequence is not a strong discriminator of the different translocation pathways, tested against multiple established hydrophobicity scales.

Taken at face value, the evidence argues that the Sec complex mostly recognizes short hydrophobic helices with a length of 8 to 13 amino acids in native proteins. Describing Sec-dependent signal peptides as “moderately hydrophobic” or “marginally hydrophobic” may subtly miss the point: The h-regions of cleaved signal peptides are generally short.

Also, most cleaved signal peptides in fungi are not recognized by the SRP. Although this conclusion is at odds with established terminology, the analysis here and elsewhere (Ast et al. 2013) has changed the field's understanding of these concepts since the time when they were named.

### Limitations of signal peptide structure prediction

The limitation of this study is that it is based on algorithmic predictions of h-regions of signal peptides, and there is little available data on the structure and function of h-regions per se. The initial identification of short hydrophobic regions as a feature of signal peptides arose from bioinformatic analyses of dozens of protein sequences (Austen 1979; von Heijne and Blomberg 1979; von Heijne 1985). The need for the h-region was confirmed by the larger datasets used to train algorithms such as Phobius and SignalP, as reviewed by Nielsen et al. (2019). However, these training data focus on predictions of overall signal peptide presence and do not validate h-region boundaries against structural data. For transmembrane helices, structural predictions are validated against an increasing number of solved structures of entire proteins and complexes (Bittrich et al. 2022; Hegedűs et al. 2022). By contrast, structural analysis of cleaved signal peptides would need to account for their function being derived through their transient interactions with the translocation machinery, before their cleavage and degradation.

The role played by the length of hydrophobic helices in targeting is supported by available structural evidence. Signal-anchors are retained hydrophobic transmembrane helices and have an average length of 24 amino acids (Saidijam et al. 2018), which is appropriate to span a lipid bilayer of width 35 to 40 Å. Structures of the translocating SRP-Sec61 complex show helices of length 17 or 19 amino acids interacting with Sec61 (PDB: 4CG6 and 3JC2) (Gogala et al. 2014; Voorhees and Hegde 2016). By contrast, the h-region of a cleaved signal peptide does not need to span a membrane, although the entire signal peptide—including flanking n-region and c-region—could be long enough to span a membrane. A structure of the active *S. cerevisiae* Sec complex includes a hydrophobic helix in the signal peptide of length ∼8 amino acids interacting with Sec61 (PDB: 7AFT) (Weng et al. 2021). Constraints on lengths of signal peptide h-regions could also arise from their cleavage later in translocation: Structures and molecular dynamics simulations indicate that short h-regions in signal peptides are specifically selected for cleavage by the human signal peptidase complex (Liaci et al. 2021).

Our analysis here uses primarily the Phobius algorithm, which predicts the overwhelming majority of fungal cleaved signal peptides to have h-regions of length 13 or less, despite the model allowing cleaved signal peptides to have length up to 20 amino acids (Käll et al. 2004). This analysis mostly aligns with predictions of overall signal peptide length from DeepTMHMM and SignalP6.0 (Fig. 4). However, these algorithms cannot be taken as ground truth; for example, Phobius does not predict h-regions of length 10, for which we cannot think of a structural explanation. SignalP version 3.0 predicted h-region lengths (Bendtsen et al. 2004) but had a strong preference for certain h-region lengths because of a specific kind of overtraining (Henrik Nielsen, personal communication). SignalP versions 4.0, 4.1, and 5.0 do not output signal peptide regions; this was reintroduced with version 6.0 (Teufel et al. 2022), but again, large-scale training data to validate h-region predictions is lacking. We found that length predictions for h-regions from SignalP6 and Phobius are poorly correlated.

To train algorithmic predictions of h-regions, studies needed include (i) further solved structures of signal peptides in translocation and peptidase complexes, (ii) molecular dynamics simulations of signal peptides in these complexes, and (iii) large-scale reporter assays on how signal peptide sequence variation affects translocation and cleavage. A template for studying structure-function relationships in signal peptides by systematic analysis of sequence variation is provided by the work of the Kendall lab in *E. coli* bacteria, reviewed by Izard and Kendall (1994).

Furthermore, experimental evidence shows that some signal peptides can be recognized by multiple translocons (Ast et al. 2013). Mutational studies show that increasing the hydrophobicity of Sec-dependent signal peptides is sufficient for them to be recognized by SRP (Ng et al. 1996; Yi et al. 2021). Some *S. cerevisiae* proteins with short but highly hydrophobic h-regions are reported as SRP-dependent (Ast et al. 2013). Conversely, reducing the hydrophobicity of signal-anchor sequences can make them Sec-dependent (Reithinger et al. 2013). Thus, although signal regions in native fungal secreted proteins have a bimodal distribution of properties corresponding to 2 major translocation routes, sequences with intermediate properties can still be translocated by one or both of the translocons. Experimental characterization of diverse and “non-standard” or non-naturally occurring sequences would be needed to understand the limits of current models.

### Differences in signal peptides between fungi and other eukaryotes

Signal sequences and translocons are not all functionally conserved across eukaryotes. Short *S. cerevisiae* signal peptides do not function in mammalian cells, such as the Sec-dependent signal peptide of yeast carboxypeptidase Y (Bird et al. 1987). Shortening the signal sequence of *S. cerevisiae* invertase maintained its secretion in yeast independent of SRP function, while these shortened invertase signal peptides were not secreted in mammalian cells (Rothe and Lehle 1998). In contrast, shortening h-regions of Sec-dependent signal peptides can lead to more efficient secretion in *S. cerevisiae (*Xue et al. 2023*)*. Another study in *S. pombe* found that a less-hydrophobic signal peptide conferred efficient secretion (Kjaerulff and Jensen 2005). The apparent preference of the fungal Sec complex for h-regions may relate to the presence of fungal-specific components on its cytoplasmic surface, homologs of Sec71 and Sec72 in *S. cerevisiae* (Schlegel et al. 2007; Rapoport et al. 2017). Even within fungi there is diversity in translocation machinery, for example, fungal homologs of Sec61 do not all complement the loss of *S. cerevisiae* Sec61 (Broughton et al. 1997; de la Rosa et al. 2004), while the SRP is not essential in *S. cerevisiae* but is essential in some other fungi (Brennwald et al. 1988; He et al. 1990). Given this diversity, it remains important to ask how results on translocation mechanisms of secreted proteins can be generalized (Delic et al. 2013).

Our analysis agrees with diverse observations arguing that the fungal Sec complex efficiently translocates signal peptides with short hydrophobic helices and that this is not exactly conserved in mammals. However, length alone is unlikely to be a complete explanation for any differences between fungal and mammalian signal peptides. One report argued that h-regions of Sec62/Sec63-dependent signal peptides in humans are longer, with a mode of 15 amino acids (Schorr et al. 2020). However, Phobius predicts equally short h-regions in human proteins as in fungi (Fig. 1). Bioinformatic analyses with SignalP 3.0 (Bendtsen et al. 2004) found similar h-region lengths of 8 to 12 or 8 to 14 aas in *S. cerevisiae* cleaved signal peptides (Xue et al. 2023) or in all experimentally verified eukaryotic signal peptides (Liaci et al. 2021), again substantially shorter than the predictions of length for transmembrane helices.

Another report argues that species-specific short motifs in signal peptides contribute to their biological activity and experimentally validated this concept in trypanosomes (Duffy et al. 2010). This suggestion is logical, given that the major functions of cleaved signal peptides involve specific interactions with the Sec complex and the signal peptidase complex, and so deserves further investigation.

### The importance of the Sec complex in fungi

Our analysis argues that secreted proteins in fungi and beyond are separated into distinct subsets that depend on distinct translocation pathways. These subsets consist of structurally and functionally distinct groups that are conserved across the fungal kingdom. In other fungi, as in *S. cerevisiae*, proteins with retained signal-anchors that are long transmembrane helices are very likely to use the SRP. By contrast, secreted cell wall proteins and others with cleaved signal peptides and hydrophobic helices are likely to use the Sec complex.

A study in *N. crassa* supports this conclusion (Liu 2017), using as model substrates 2 proteins with cleaved signal peptides, CBH-1 and the N-terminal region of ASD-1, each fused to an ER-retained fluorescent protein (roGFP-KDEL). They observed Sec-dependent translocation of CBH1-roGFP-KDEL, which has an h-region of predicted length 8. By contrast, they observed SRP-dependent translocation of ASD-1(N32)-roGFP-KDEL, which has an h-region of predicted length 16 and higher hydrophobicity. Although this study uses only 2 substrates, it supports the idea that h-region length determines translocation route. This was the only direct experimental test of Sec-dependent or SRP-dependent translocation that we were able to find in fungi beyond *S. cerevisiae*.

The regulation and function of the Sec complex is likely to be critical for cell wall biogenesis and protein secretion across fungi. Secreted proteins with cleaved signal peptides include major secreted virulence factors in fungal pathogens of plants and animals, such as *C. albicans* candidalysin and secreted aspartyl proteases (Brunke et al. 2016). Predicted cleaved signal peptides are already used to computationally identify candidate secreted effector proteins in plant fungal pathogens that might suppress host immunity or modify host cellular activities (Derbyshire and Raffaele 2023; Seong and Krasileva 2023). Indeed, computational screens of conserved signal peptide-containing proteins can be an efficient way to discover virulence factors (Schuster et al. 2024). Thus, investigating fungal secretion mechanisms, including translocation routes and their regulation, will be critical to understand fungal pathogenesis.

Further work is needed to experimentally validate our computational predictions. In *S. cerevisiae*, partial loss-of-function mutants of Sec62, Sec63, and the non-essentiality of Sec71 and Sec72 facilitated genetic analysis of the Sec complex and its substrates (Ng et al. 1996). The core results, that the Sec complex promotes secretion of proteins with cleaved signal peptides in *S. cerevisiae*, were validated by a combination of large-scale genetic screens, functional genomics, and detailed experiments with signal peptide swaps in reporter genes (Ast et al. 2013; Jan et al. 2014; Costa et al. 2018; Yim et al. 2018; Yi et al. 2021). Large-scale experiments in diverged fungi are needed to directly measure the substrates of alternative secretion pathways. We speculate that there may be yet more complexity in secretion pathways in other fungi, especially in early-diverging fungi and in multicellular fungi with complex lifestyles and large secretomes.

## Supplementary Material

jkag134_Supplementary_Data

## Data Availability

All data, data analysis code, and figures are available at https://github.com/TristanSones-Dykes/TMSP_Pub (doi:10.5281/zenodo.19801296). Supplemental material available at G3 online.
