# Supplementary material for: Protein secretion routes in fungi are predicted by the length of the hydrophobic helix in the signal sequence
Source: G3 (Bethesda). 2026 Jul 10;16(8):jkag134. doi: 10.1093/g3journal/jkag134 (PMC13439945; doi:10.1093/g3journal/jkag134)
Supplement: Supplementary file 1 [file jkag134_supplementary_data.pdf]

Protein secretion routes in fungi are predicted by the length of the hydrophobic helix in the signal sequence

Tristan Sones-Dykes, Edward Wallace

G3: Genes | Genomes |Genetics, 2026

Supplementary figures S1 to S4

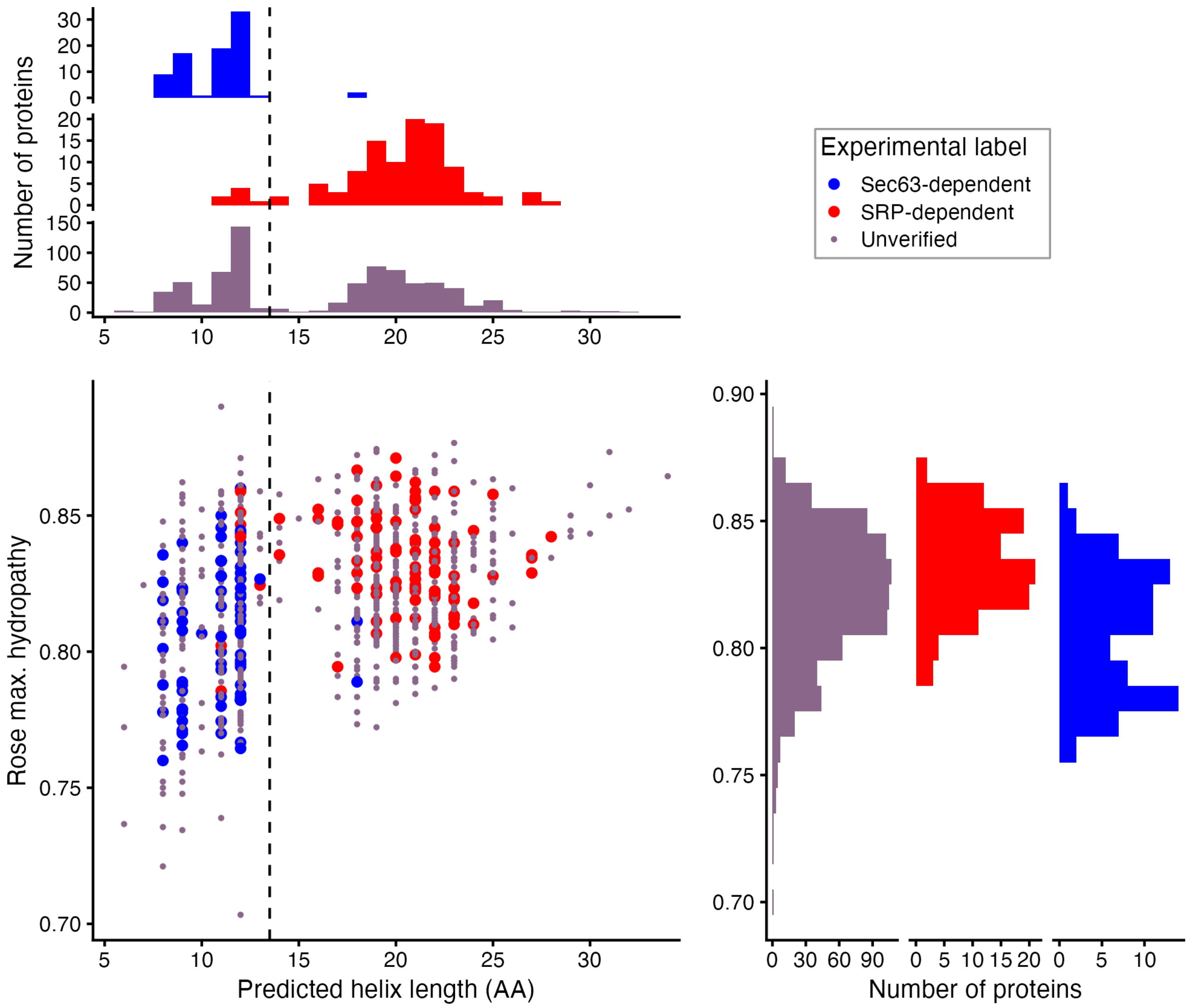

Figure S1. Hydrophobic helices in signal regions in *S. cerevisiae*, have a wider distribution of maximum hydropathy calculated on the Rose scale. This figure is similar to Figure 2, except calculating the Rose hydrophobicity (Rose et al. 1985) of a 9 aa window (y axis) instead of the Kyte-Doolittle hydrophobicity.

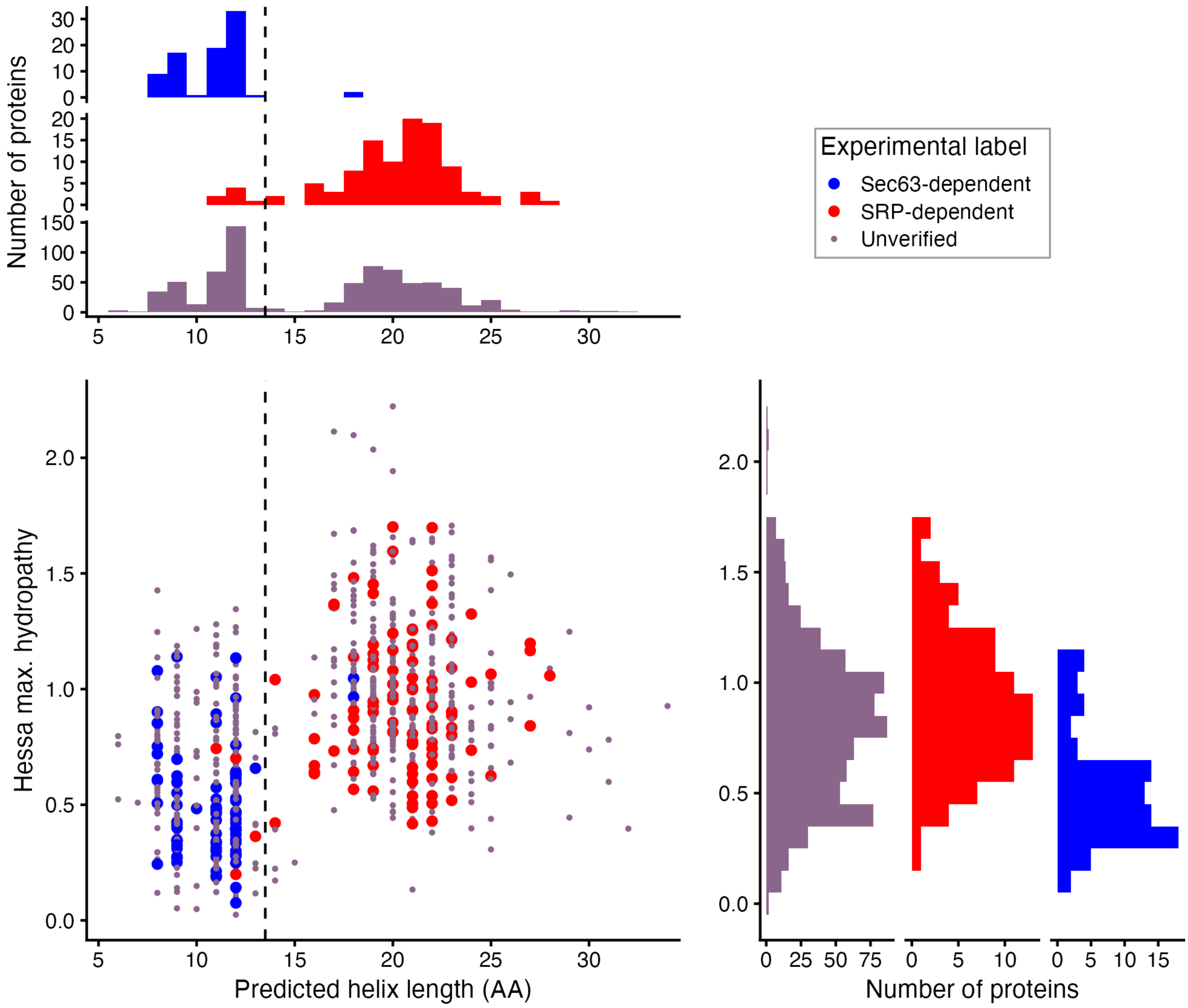

Figure S2. Hydrophobic helices in signal regions in *S. cerevisiae*, have a wider distribution of maximum hydropathy calculated on the Hessa scale. This figure is similar to Figure 2, however calculated using the Hessa hydrophobicity scale (Hessa et al. 2005) of a 9 aa window (y axis) instead of the Kyte-Doolittle hydrophobicity.

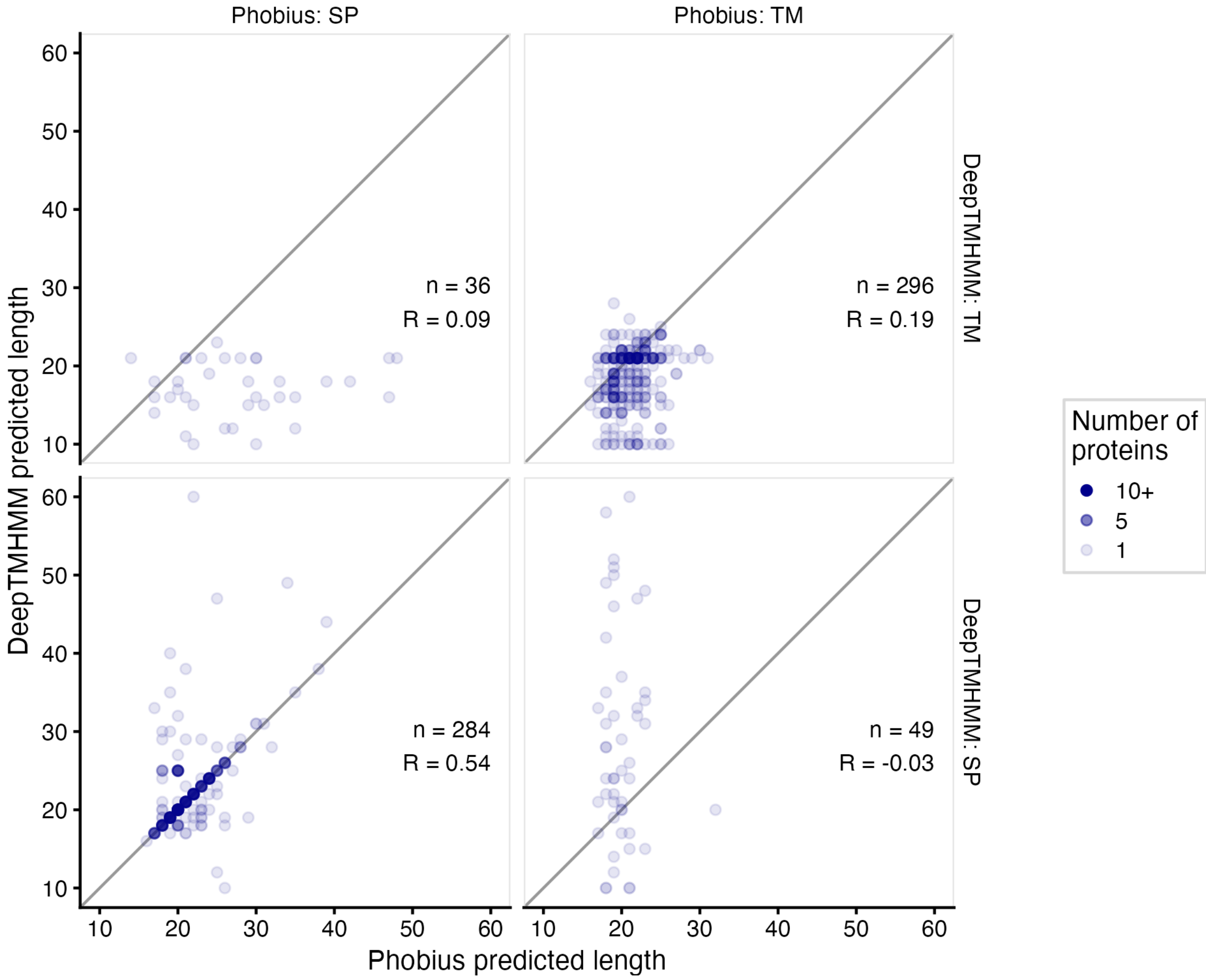

Figure S3. Two computational tools, DeepTMHMM and Phobius, mostly agree on their predictions of secretion signal categories and lengths. Each panel compares the predicted lengths of the entire signal sequence from *S. cerevisiae* proteins, restricted to *S. cerevisiae* proteins for which either tool predicted such features in the initial 60 amino acids. The opacity of each bubble is proportion to the count of proteins with that combination of predicted lengths. Panels are separated by being predicted as either cleaved signal peptide (SP) or retained signal-anchor transmembrane region (TM). This figure shows the length of the entire signal peptide, which is longer than the hydrophobic h-region, because DeepTMHMM outputs only the overall length of the signal peptide. Note that the bottom left panel of this Figure S3 shows the same data as the corresponding panel in Figure 4.

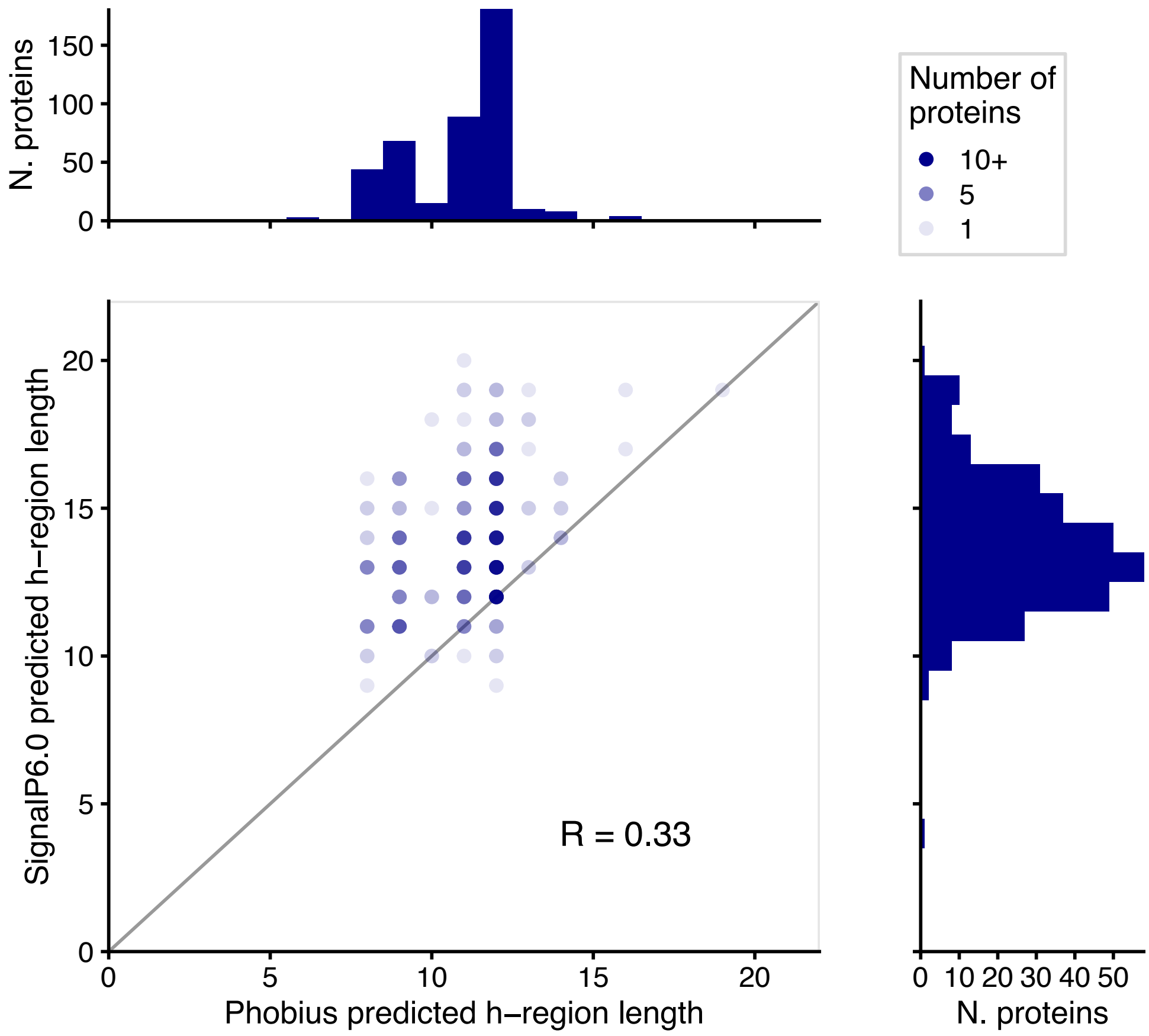

Figure S4. Two computational tools, SignalP6.0 and Phobius, weakly agree on their predictions of h-region lengths in cleaved signal peptides. Each panel compares the predicted lengths of the h-region from *S. cerevisiae* proteins for which both tools predict cleaved signal peptides. The opacity of each bubble is proportion to the count of proteins with that combination of predicted lengths.
